# Crystal structure of poly[(μ_3_-4-amino-1,2,5-oxa­diazole-3-hydroxamato)thallium(I)]

**DOI:** 10.1107/S2056989020001577

**Published:** 2020-02-11

**Authors:** Inna S. Safyanova, Oksana A. Bondar, Anna V. Pavlishchuk, Iryna V. Omelchenko, Turganbay S. Iskenderov, Valentina A. Kalibabchuk

**Affiliations:** a Department of Chemistry, National Taras Shevchenko University of Kyiv, Volodymyrska Street 64, Kyiv, 01601, Ukraine; b SSI "Institute for Single Crystals", National Academy of Sciences of Ukraine, Nauky ave. 60, 61001 Kharkiv, Ukraine; c Department of General Chemistry, O.O. Bohomolets National Medical University, Shevchenko Blvd. 13, 01601 Kyiv, Ukraine

**Keywords:** crystal structure, 1,2,5-oxa­diazole, hydroxamic acid, thallium(I), tautomerism

## Abstract

The title compound represents the thallium(I) salt of a substituted 1,2,5-oxa­diazole, [Tl(C_3_H_3_N_4_O_3_)]_*n*_, with amino- and hydroxamate groups in the 4- and 3- positions of the oxa­diazole ring, respectively. In the crystal, the deprotonated hydroxamate group represents an inter­mediate between the keto/enol tautomers and forms a five-membered chelate ring with the thallium(I) cation. The coordination sphere of the cation is augmented to a distorted disphenoid by two monodentately binding O atoms from two adjacent anions, leading to the formation of zigzag chains extending parallel to the *b* axis. The cohesion within the chains is supported by π–π stacking [centroid–centroid distance = 3.746 (3) Å] and inter­molecular N—H⋯N hydrogen bonds.

## Abstract 2

The thallium(I) atom in the polymeric title compound is bonded to four O atoms, with the lone pair electrons stereochemically active.

## Chemical context   

Substituted oxa­diazo­les attract attention because of their wide range of applications in organic synthesis as useful inter­mediates (Romeo & Chiacchio, 2011 ▸; Zlotin *et al.*, 2017 ▸) and for drug design (Giorgis *et al.*, 2011 ▸; Pal *et al.*, 2017 ▸; Stepanov *et al.*, 2015 ▸). In addition, mol­ecules with the oxa­diazole moiety can be considered for the creation of energetic systems (Zhang *et al.*, 2015 ▸) with high thermal stability and mechanical sensitivity. The variety of coordination modes typical for oxa­diazole-containing ligands result in the formation of multiple mono- and polynuclear complexes, as well as coord­ination polymers (Akhbari & Morsali, 2010 ▸). Complexes with oxa­diazole-based ligands have demonstrated significant biological activity as anti-cancer (Glomb *et al.*, 2018 ▸), anti-inflammatory (Singh *et al.*, 2013 ▸), anti-tuberculosis (De *et al.*, 2019 ▸) and anti-malarial (Zareef *et al.*, 2007 ▸) agents.

However, the standard synthetic procedures for oxa­diazole-containing scaffolds usually utilizes the dehydrative cyclization of bis-oximes, which is performed at high temperatures (Fershtat & Makhova, 2016 ▸; Romeo & Chiacchio, 2011 ▸) and often includes the introduction of different activating reagents (Shaposhnikov *et al.*, 2003 ▸; Telvekar & Takale, 2013 ▸). A convenient procedure for the synthesis of substituted 4-amino-1,2,5-oxa­diazo­les based on the formation of bis-oximes *in situ* from the hydroxyl­amine and cyano-oximes was recently proposed (Neel & Zhao, 2018 ▸). The introduction of dehydrating agents allows a significant decrease in the temperature during reaction, gave the possibility to synthesize substituted 1,2,5-oxa­diazo­les with various side functional groups. In this regard, we have adapted the synthetic procedure for 1,2,5-oxa­diazole with amino- and hydroxamate groups in the 4- and 3- position of the 1,2,5-oxa­diazole ring, respectively, and report here the thallium(I) salt of this compound, **1**, Tl(C_3_H_3_N_4_O_3_). The introduction of a hydroxamic group at the 1,2,5-oxa­diazole ring allows the consideration of potentially inter­esting ligand systems for the synthesis of various polynuclear complexes (Pavlishchuk *et al.*, 2018 ▸; Lutter *et al.*, 2018 ▸; Ostrowska *et al.*, 2019 ▸; Gumienna-Kontecka *et al.*, 2007 ▸).
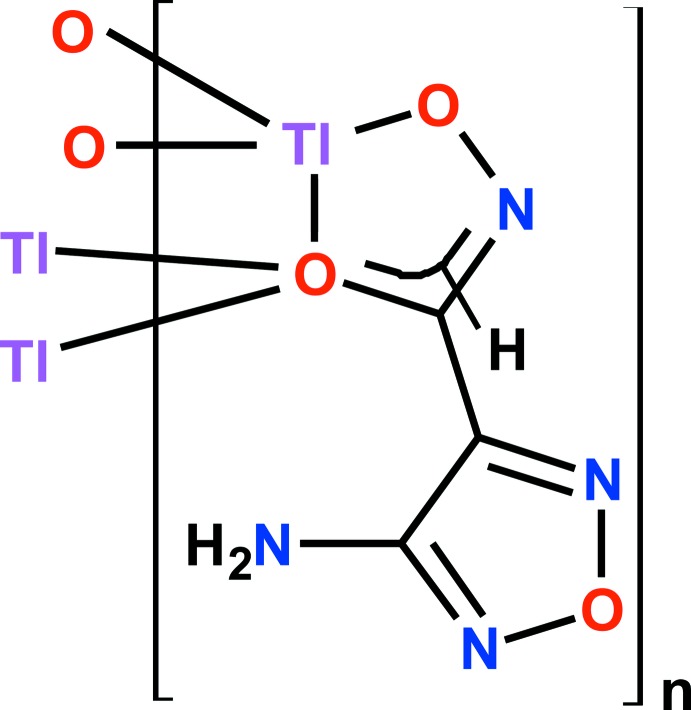


## Structural commentary   

The asymmetric unit of **1** comprises one 4-amino-1,2,5-oxa­diazole-3 hydroxamate anion and a thallium(I) cation. The oxa­diazole ring C2/C3/N2/O3/N3 is almost planar with the largest deviation from the least-squares plane being 0.007 Å for C2. The C2=N2 and C3=N3 bond lengths [1.304 (14) and 1.329 (11) Å, respectively] are typical for C=N double bonds in substituted oxa­diazole cycles (Viterbo & Serafino, 1978 ▸), and the N2—O3 and N3—O3 bonds [1.365 (11) and 1.419 (11) Å, respectively] also fall in a range typical for 1,2,5-oxa­diazo­les (Fonari *et al.*, 2003 ▸; Viterbo & Serafino, 1978 ▸). The substituent amino- and hydroxamate groups in the 4- and 3- positions, respectively, of the 1,2,5-oxa­diazole ring are nearly coplanar with the oxa­diazole ring, with a deviation of 0.071 Å for nitro­gen atom N4 of the amino group and a dihedral angle between the mean plane of the heterocycle and the hydroxamate group C1/O2/N1/O1 of 8.4 (4)°. The C3—N4 [1.360 (13) Å] and N1—O1 [1.412 (9) Å] bond lengths are typical for a non-coordinating amino group (Fonari *et al.*, 2003 ▸; Viterbo & Serafino, 1978 ▸) and for a deprotonated hydroxamate group (Golenya *et al.*, 2012 ▸; Safyanova *et al.*, 2017 ▸), respectively. On the other hand, the C1—N1 [1.314 (12) Å] and C1—O2 [1.275 (11) Å] bond lengths are inter­mediate between the tautomeric keto and enol forms (Larsen, 1988 ▸), accompanied by a delocalization of the π electrons over the N1—C1—O2 backbone and a disorder of the corresponding hydrogen atom that could not be localized from difference-Fourier maps.

**Figure 1:**
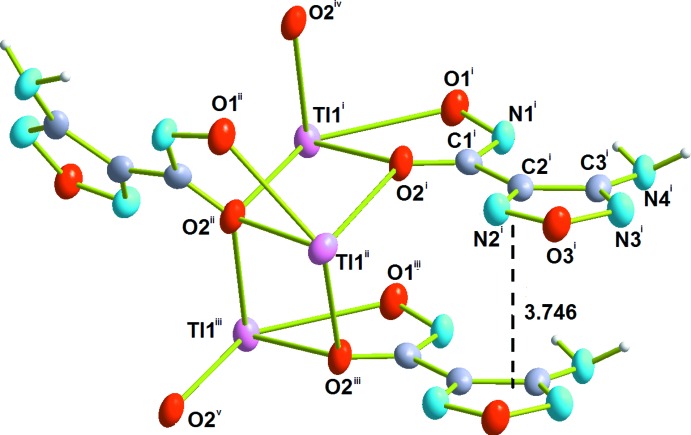
A fragment of the crystal structure of **1** showing the coordination environment of the Tl1 ions with displacement ellipsoids drawn at the 50% probability level**.** [Symmetry codes: (i) 1 + *x*, *y*, *z*; (ii) 1 − *x*, 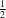 + *y*, 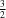 − *z*; (iii) 1 + *x*, 1 + *y*, *z*; (iv) 1 − *x*, −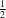 + *y*, 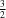 − *z*; (v) 1 − *x*, 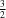 + *y*, 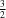 − *z*.]

**Figure 2:**
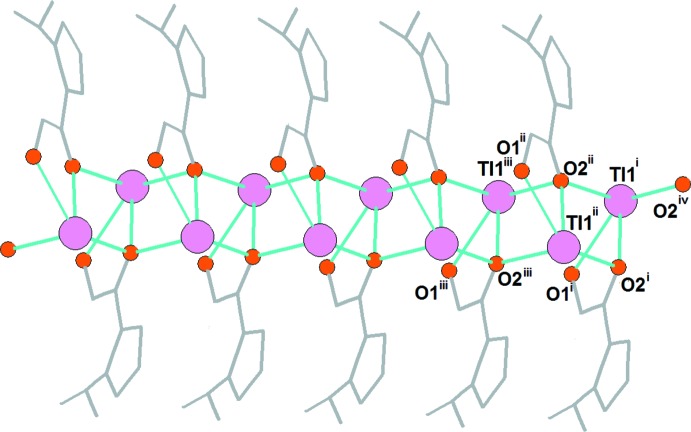
The formation of polymeric zigzag chains in **1.** [Symmetry codes: (i) 1 + *x*, *y*, *z*; (ii) 1 − *x*, 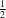 + *y*, 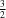 − *z*; (iii) 1 + *x*, 1 + *y*, *z*; (iv) 1 − *x*, −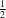 + *y*, 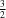 − *z*; (v) 1 − *x*, 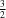 + *y*, 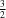 − *z*.]

The Tl1 cation in **1** is bonded to the bidentate hydroxamate anion through oxygen atoms O1 [2.814 (7) Å] and O2 [2.537 (7) Å] in the form of a five-membered chelate ring. The coordination sphere of the Tl1 cation in **1** is augmented to four by two monodentately binding O2 atoms of two adjacent oxa­diazole moieties with distances of Tl1—O2^ii^ = 2.880 (7) Å and Tl1—O2^i^ = 2.761 (7) Å [symmetry codes: (i) −*x*, *y* + 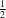, −*z* + 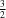; (ii) −*x*, *y* − 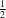, −*z* + 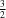] (Fig. 1 ▸). The bond length Tl1—O2 is *ca* 0.2–0.3 Å shorter in the case of the chelating coordination mode of the hydroxamate group compared with the monodentate coordination mode. Thus, each O2 atom is involved in a chelate coordination with one Tl1 ion and in a monodentate coordination with two other Tl1 ions, forming zigzag chains extending along the *b-*axis direction (Fig. 2 ▸). The Tl—O bond lengths involving the hydroxamate oxygen atoms in **1** are typical for Tl^I^ compounds (Salassa & Terenzi, 2019 ▸), and the formation of similar polymeric chains is frequently observed for Tl^I^ complexes (Akhbari *et al.*, 2009 ▸). The resulting coordination sphere of Tl1 can be best described as a distorted seesaw (SS-4) or disphenoid with a stereochemically active lone pair (Mudring & Rieger, 2005 ▸). If longer bonds are taken into account (Akhbari & Morsali, 2010 ▸; Schroffenegger *et al.*, 2020 ▸), the Tl1 cation also has weak inter­actions at 3.453 (8), 3.289 (9), 3.385 (7) and 3.219 (8) Å with O3^iv^, N2^ii^, O1^v^ and O3^vi^ [symmetry codes: (iv) *x*, −*y* + 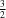, *z* + 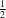; (v) *x*, *y* + 1, *z*; (vi) *x*, −*y* + 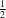, *z* + 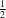] atoms from another three oxa­diazole moieties. The closest contact between adjacent Tl1 cations within a zigzag chain is 3.7458 (5) Å.

## Supra­molecular features   

**Figure 3:**
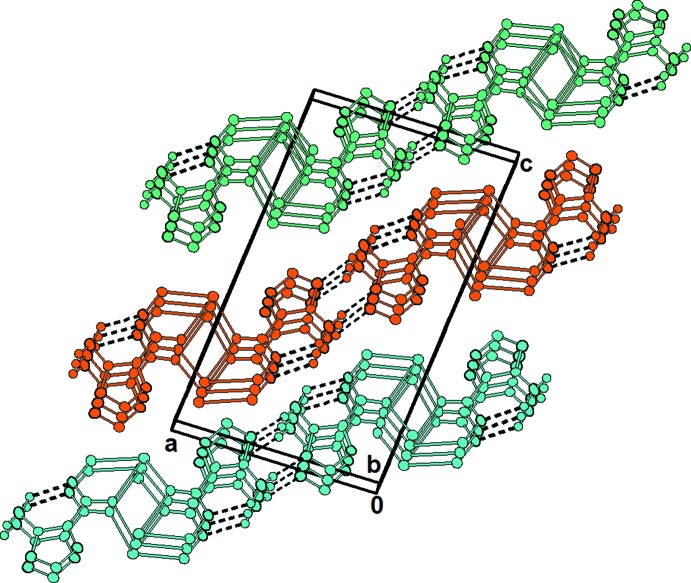
Packing diagram of **1**, with hydrogen bonds indicated by dashed lines.

In the crystal, the oxa­diazole rings are stacked in a parallel manner with a centroid–centroid distance = 3.746 (3) Å (Fig. 1 ▸). Together with weak inter­molecular hydrogen bonds between the amino group (N4) and two nitro­gen atoms from the azolo (N3) and the hydroxamic (N1) group (Table 1 ▸, Fig. 3 ▸) they support the cohesion of the chains along the *b-*axis direction.

## Database survey   

A search in the Cambridge Structural Database (CSD version 5.39, update of May 2018; Groom *et al.*, 2016 ▸) for substituted oxa­diazo­les revealed two structures, *viz.* 3-amino-4-methyl­furazan (Pibiri *et al.*, 2018 ▸) and 4-amino-1,2,5-oxa­diazole-3-carboxamide oxime (Zhang & Jian, 2009 ▸). Tl^I^ complexes with comparable organic ligands have been reported for thallium (anthrano­yl)anthranilate (Wiesbrock & Schmidbaur, 2004 ▸), thallium(I) 2-amino-benzoate (Wiesbrock & Schmidbaur, 2003 ▸), thallium(I) aryl­cyanoxime (Robertson *et al.*, 2004 ▸) [Tl_4_(H_2_O)_2_(anthracene-9-carboxyl­ate)_4_] (Kumar *et al.*, 2015 ▸), bis­[(*μ*-1,3-di­phenyl­propane-1,3-dionato-*O*,*O*′:*O*′)di­methylthallium] (Britton, 2001 ▸) and thallium(I) 4-hy­droxy­benzyl­idene-4-amino­benzoate (Akhbari *et al.*, 2009 ▸).

## Synthesis and crystallization   

**Figure 4:**
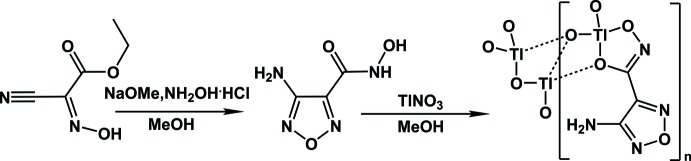
Synthesis scheme for 4-amino-1,2,5-oxa­diazole-3 hydroxamate thallium(I).

The title compound was obtained according to a modification of the procedure reported by Neel & Zhao (2018 ▸) (Fig. 4 ▸). Solutions containing 5 mmol of hydroxyl­amine hydro­chloride in 10 ml of methanol, and 10 mmol of sodium methoxide in 15 ml of methanol were stirred for 30 min while cooling in an ice bath. The formed precipitate of sodium chloride was filtered off. The methano­lic solutions of ethyl-2-cyano-2-(hy­droxy­imino)­acetate (5 mmol) and hydroxyl­amine were combined and stirred for 5 h at room temperature. The resulting white precipitate was filtered off and dissolved in 5 ml of water, followed by HCl addition to pH = 5. The organic compound was extracted with ethyl acetate; the extract was subsequently dried over anhydrous Na_2_SO_4_, and the solvent was finally removed by rotary evaporation. Colorless crystals of **1** suitable for single crystal X-ray analysis were obtained by combining the organic compound with thallium(I) nitrate in iso­propanol and subsequent slow evaporation of the solvent at ambient temperature within 48 h (yield 16.5%).

## Refinement   

Crystal data, data collection and structure refinement details are summarized in Table 2 ▸. The H atoms of the amino group were located from a difference-Fourier map; their coordinates were refined freely with *U*_iso_(H) = 1.2*U*_eq_(N). The hydrogen atom of the hydroxamate function could not be observed in difference-Fourier maps, and a tentative calculated position was in too close vicinity to atom H4*B* of the amino group. Most probably, the hydroxamate H atom is disordered over the N1—C1—O2 backbone due to the presence of both tautomeric forms. Hence, this H atom is not included in the final model. The highest remaining electron density is located 0.88 Å from Tl1.

## Supplementary Material

Crystal structure: contains datablock(s) I. DOI: 10.1107/S2056989020001577/wm5530sup1.cif

Structure factors: contains datablock(s) I. DOI: 10.1107/S2056989020001577/wm5530Isup2.hkl

CCDC reference: 1981874

Additional supporting information:  crystallographic information; 3D view; checkCIF report

## Figures and Tables

**Table 1:** Hydrogen-bond geometry (Å, °)

*D*—H⋯*A*	*D*—H	H⋯*A*	*D*⋯*A*	*D*—H⋯*A*
N4—H4*A*⋯N3^i^	0.93	2.23	3.156 (10)	169
N4—H4*B*⋯N1^ii^	1.01	2.65	3.256 (13)	118

Symmetry codes: (i) 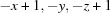; (ii) 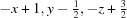.

**Table 2:** Experimental details

Crystal data	
Chemical formula	[Tl(C_3_H_3_N_4_O_3_)]
*M*_r_	347.46
Crystal system, space group	Monoclinic, *P*2_1_/*c*
Temperature (K)	298
*a*, *b*, *c* (Å)	10.0731 (4), 3.74576 (18), 16.9805 (6)
β (°)	95.808 (4)
*V* (Å^3^)	637.41 (5)
*Z*	4
Radiation type	Mo *K*α
μ (mm^−1^)	25.30
Crystal size (mm)	0.2 × 0.2 × 0.2

Data collection	
Diffractometer	Agilent Xcalibur Sapphire3 CCD
Absorption correction	Multi-scan (*CrysAlis PRO*; Agilent, 2012 ▸)
*T*_min_, *T*_max_	0.231, 1.000
No. of measured, independent and observed [*I* > 2σ(*I*)] reflections	4634, 1452, 1320
*R*_int_	0.056
(sin θ/λ)_max_ (Å^−1^)	0.650

Refinement	
*R*[*F* ^2^ > 2σ(*F* ^2^)], *wR*(*F* ^2^), *S*	0.045, 0.111, 1.07
No. of reflections	1452
No. of parameters	100
H-atom treatment	H-atom parameters constrained
Δρ_max_, Δρ_min_ (e Å^−3^)	6.17, −2.23

Computer programs: *CrysAlis PRO* (Agilent, 2012 ▸), *SHELXT* (Sheldrick, 2015 ▸), *olex2.refine* (Bourhis *et al.*, 2015 ▸) and *OLEX2* (Dolomanov *et al.*, 2009 ▸).

## supplementary crystallographic information

### Crystal data

**Table:**

[Tl(C_3_H_3_N_4_O_3_)]	*F*(000) = 612
*M**_r_* = 347.46	*D*_x_ = 3.610 Mg m^−^^3^
Monoclinic, *P*2_1_/*c*	Mo *K*α radiation, λ = 0.71073 Å
*a* = 10.0731 (4) Å	Cell parameters from 216 reflections
*b* = 3.74576 (18) Å	θ = 4.4–22.3°
*c* = 16.9805 (6) Å	µ = 25.30 mm^−^^1^
β = 95.808 (4)°	*T* = 298 K
*V* = 637.41 (5) Å^3^	Block, clear colourless
*Z* = 4	0.2 × 0.2 × 0.2 mm

### Data collection

**Table:**

Agilent Xcalibur Sapphire3 CCD diffractometer	1320 reflections with *I* > 2σ(*I*)
ω scans	*R*_int_ = 0.056
Absorption correction: multi-scan (*CrysAlis PRO*; Agilent, 2012)	θ_max_ = 27.5°, θ_min_ = 3.0°
*T*_min_ = 0.231, *T*_max_ = 1.000	*h* = −13→13
4634 measured reflections	*k* = −4→4
1452 independent reflections	*l* = −22→19

### Refinement

**Table:**

Refinement on *F*^2^	0 restraints
Least-squares matrix: full	Hydrogen site location: difference Fourier map
*R*[*F*^2^ > 2σ(*F*^2^)] = 0.045	H-atom parameters constrained
*wR*(*F*^2^) = 0.111	*w* = 1/[σ^2^(*F*_o_^2^) + (0.0629*P*)^2^] where *P* = (*F*_o_^2^ + 2*F*_c_^2^)/3
*S* = 1.07	(Δ/σ)_max_ = 0.001
1452 reflections	Δρ_max_ = 6.17 e Å^−^^3^
100 parameters	Δρ_min_ = −2.23 e Å^−^^3^

### Special details

**Table:**

Geometry. All esds (except the esd in the dihedral angle between two l.s. planes) are estimated using the full covariance matrix. The cell esds are taken into account individually in the estimation of esds in distances, angles and torsion angles; correlations between esds in cell parameters are only used when they are defined by crystal symmetry. An approximate (isotropic) treatment of cell esds is used for estimating esds involving l.s. planes.

### Fractional atomic coordinates and isotropic or equivalent isotropic displacement parameters (Å^2^)

**Table:**

	*x*	*y*	*z*	*U*_iso_*/*U*_eq_	
Tl1	0.05880 (4)	0.45183 (11)	0.86058 (2)	0.03141 (19)	
O2	0.1164 (7)	0.4279 (17)	0.7187 (4)	0.0298 (15)	
O1	0.2851 (8)	0.0779 (19)	0.8210 (4)	0.0339 (17)	
O3	0.2375 (8)	0.4924 (18)	0.4946 (5)	0.0339 (17)	
N2	0.1809 (10)	0.510 (2)	0.5642 (6)	0.0299 (19)	
N1	0.3245 (8)	0.148 (2)	0.7450 (4)	0.0301 (17)	
C1	0.2298 (9)	0.319 (2)	0.7013 (5)	0.0257 (18)	
N4	0.4966 (9)	0.110 (2)	0.6207 (5)	0.0339 (19)	
H4A	0.528766	−0.041040	0.583376	0.041*	
H4B	0.482666	0.024160	0.675476	0.041*	
N3	0.3648 (8)	0.328 (2)	0.5070 (4)	0.0332 (18)	
C2	0.2653 (9)	0.367 (3)	0.6185 (5)	0.0267 (18)	
C3	0.3828 (9)	0.254 (2)	0.5839 (5)	0.0243 (17)	

### Atomic displacement parameters (Å^2^)

**Table:**

	*U*^11^	*U*^22^	*U*^33^	*U*^12^	*U*^13^	*U*^23^
Tl1	0.0281 (3)	0.0419 (3)	0.0240 (3)	0.00530 (14)	0.00185 (18)	−0.00084 (13)
O2	0.019 (3)	0.047 (4)	0.024 (3)	0.002 (3)	0.004 (3)	−0.002 (3)
O1	0.026 (4)	0.053 (4)	0.023 (4)	0.001 (3)	0.004 (3)	0.006 (3)
O3	0.030 (4)	0.049 (4)	0.023 (4)	0.001 (3)	0.002 (3)	0.003 (3)
N2	0.028 (5)	0.040 (4)	0.022 (4)	0.004 (3)	0.003 (4)	0.003 (3)
N1	0.027 (4)	0.041 (4)	0.023 (4)	0.005 (4)	0.005 (3)	0.002 (3)
C1	0.032 (5)	0.024 (4)	0.022 (4)	−0.002 (4)	0.003 (4)	−0.004 (3)
N4	0.031 (5)	0.044 (5)	0.029 (4)	0.008 (4)	0.011 (4)	−0.001 (4)
N3	0.028 (4)	0.042 (5)	0.028 (4)	0.009 (4)	0.000 (3)	0.002 (4)
C2	0.020 (4)	0.029 (4)	0.030 (5)	−0.002 (4)	−0.001 (4)	−0.005 (4)
C3	0.023 (4)	0.030 (4)	0.020 (4)	−0.004 (3)	0.000 (3)	−0.006 (3)

### Geometric parameters (Å, º)

**Table:**

Tl1—O2	2.537 (7)	O1—N1	1.412 (9)
Tl1—O2^i^	2.761 (7)	O3—N2	1.365 (11)
Tl1—O1	2.814 (7)	O3—N3	1.419 (11)
Tl1—O2^ii^	2.880 (7)	N2—C2	1.304 (14)
Tl1—O3^iii^	3.219 (8)	N1—C1	1.314 (12)
Tl1—N2^ii^	3.289 (9)	C1—C2	1.497 (11)
Tl1—C1^i^	3.291 (9)	N4—C3	1.360 (13)
Tl1—O1^iv^	3.385 (7)	N4—H4A	0.9324
Tl1—C1	3.387 (8)	N4—H4B	1.0077
Tl1—O3^v^	3.453 (8)	N3—C3	1.329 (11)
O2—C1	1.275 (11)	C2—C3	1.437 (11)
			
O2—Tl1—O2^i^	75.9 (2)	N1—O1—Tl1^vi^	124.6 (6)
O2—Tl1—O1	59.1 (2)	Tl1—O1—Tl1^vi^	73.70 (15)
O2^i^—Tl1—O1	134.3 (2)	N1—O1—Tl1^ii^	69.9 (5)
O2—Tl1—O2^ii^	73.8 (2)	Tl1—O1—Tl1^ii^	67.89 (16)
O2^i^—Tl1—O2^ii^	83.2 (2)	Tl1^vi^—O1—Tl1^ii^	64.47 (13)
O1—Tl1—O2^ii^	91.3 (2)	N2—O3—N3	110.1 (8)
O2—Tl1—O3^iii^	119.2 (2)	N2—O3—Tl1^vii^	112.7 (6)
O2^i^—Tl1—O3^iii^	164.3 (2)	N3—O3—Tl1^vii^	108.2 (5)
O1—Tl1—O3^iii^	60.21 (19)	N2—O3—Tl1^viii^	107.8 (5)
O2^ii^—Tl1—O3^iii^	104.50 (19)	N3—O3—Tl1^viii^	139.7 (5)
O2—Tl1—O1^iv^	67.2 (2)	Tl1^vii^—O3—Tl1^viii^	68.20 (17)
O2^i^—Tl1—O1^iv^	82.28 (19)	N2—O3—Tl1^i^	29.7 (5)
O1—Tl1—O1^iv^	73.70 (16)	N3—O3—Tl1^i^	137.7 (5)
O2^ii^—Tl1—O1^iv^	140.56 (18)	Tl1^vii^—O3—Tl1^i^	103.6 (2)
O3^iii^—Tl1—O1^iv^	99.15 (19)	Tl1^viii^—O3—Tl1^i^	78.16 (14)
O2—Tl1—O3^v^	119.6 (2)	N2—O3—Tl1^ii^	36.6 (5)
O2^i^—Tl1—O3^v^	101.3 (2)	N3—O3—Tl1^ii^	111.1 (5)
O1—Tl1—O3^v^	94.4 (2)	Tl1^vii^—O3—Tl1^ii^	78.51 (15)
O2^ii^—Tl1—O3^v^	166.55 (18)	Tl1^viii^—O3—Tl1^ii^	107.4 (2)
O3^iii^—Tl1—O3^v^	68.20 (17)	Tl1^i^—O3—Tl1^ii^	49.53 (8)
O1^iv^—Tl1—O3^v^	52.89 (17)	C2—N2—O3	107.0 (8)
Tl1—O2—Tl1^ii^	106.8 (2)	C1—N1—O1	110.6 (7)
C1—O2—Tl1^i^	129.1 (6)	O2—C1—N1	129.9 (8)
Tl1—O2—Tl1^i^	103.4 (2)	O2—C1—C2	118.9 (9)
Tl1^ii^—O2—Tl1^i^	83.2 (2)	N1—C1—C2	111.1 (7)
C1—O2—Tl1^vi^	91.8 (5)	C3—N4—H4A	105.2
Tl1—O2—Tl1^vi^	57.29 (13)	C3—N4—H4B	111.1
Tl1^ii^—O2—Tl1^vi^	67.75 (13)	H4A—N4—H4B	121.6
Tl1^i^—O2—Tl1^vi^	134.9 (2)	C3—N3—O3	105.6 (6)
C1—O2—Tl1^iv^	123.5 (6)	N2—C2—C3	109.7 (8)
Tl1—O2—Tl1^iv^	54.51 (12)	N2—C2—C1	120.8 (8)
Tl1^ii^—O2—Tl1^iv^	133.3 (2)	C3—C2—C1	129.3 (9)
Tl1^i^—O2—Tl1^iv^	64.69 (12)	N3—C3—N4	124.0 (7)
Tl1^vi^—O2—Tl1^iv^	111.80 (14)	N3—C3—C2	107.6 (8)
N1—O1—Tl1	115.8 (5)	N4—C3—C2	128.3 (8)
			
N3—O3—N2—C2	−0.1 (10)	Tl1^vi^—N1—C1—Tl1^i^	74.4 (19)
Tl1^vii^—O3—N2—C2	120.8 (7)	O1—N1—C1—Tl1^vi^	−38.2 (6)
Tl1^viii^—O3—N2—C2	−166.0 (6)	Tl1—N1—C1—Tl1^vi^	−48.4 (2)
Tl1^i^—O3—N2—C2	−161.9 (14)	Tl1^ii^—N1—C1—Tl1^vi^	29.4 (5)
Tl1^ii^—O3—N2—C2	98.6 (9)	N2—O3—N3—C3	−0.6 (10)
N3—O3—N2—Tl1^i^	161.8 (7)	Tl1^vii^—O3—N3—C3	−124.1 (6)
Tl1^vii^—O3—N2—Tl1^i^	−77.3 (8)	Tl1^viii^—O3—N3—C3	158.3 (6)
Tl1^viii^—O3—N2—Tl1^i^	−4.1 (10)	Tl1^i^—O3—N3—C3	12.7 (11)
Tl1^ii^—O3—N2—Tl1^i^	−99.5 (12)	Tl1^ii^—O3—N3—C3	−39.7 (8)
N3—O3—N2—Tl1^ii^	−98.7 (9)	N2—O3—N3—Tl1^vii^	123.5 (7)
Tl1^vii^—O3—N2—Tl1^ii^	22.2 (9)	Tl1^viii^—O3—N3—Tl1^vii^	−77.5 (7)
Tl1^viii^—O3—N2—Tl1^ii^	95.4 (6)	Tl1^i^—O3—N3—Tl1^vii^	136.8 (8)
Tl1^i^—O3—N2—Tl1^ii^	99.5 (12)	Tl1^ii^—O3—N3—Tl1^vii^	84.4 (3)
N3—O3—N2—Tl1^vii^	−120.9 (7)	N2—O3—N3—Tl1^viii^	−158.9 (12)
Tl1^viii^—O3—N2—Tl1^vii^	73.2 (4)	Tl1^vii^—O3—N3—Tl1^viii^	77.5 (7)
Tl1^i^—O3—N2—Tl1^vii^	77.3 (8)	Tl1^i^—O3—N3—Tl1^viii^	−145.6 (13)
Tl1^ii^—O3—N2—Tl1^vii^	−22.2 (9)	Tl1^ii^—O3—N3—Tl1^viii^	161.9 (10)
N3—O3—N2—Tl1^viii^	165.9 (8)	O3—N2—C2—C3	0.7 (11)
Tl1^vii^—O3—N2—Tl1^viii^	−73.2 (4)	Tl1^i^—N2—C2—C3	−166.4 (6)
Tl1^i^—O3—N2—Tl1^viii^	4.1 (10)	Tl1^ii^—N2—C2—C3	131.4 (7)
Tl1^ii^—O3—N2—Tl1^viii^	−95.4 (6)	Tl1^vii^—N2—C2—C3	52.0 (10)
Tl1—O1—N1—C1	−13.7 (9)	Tl1^viii^—N2—C2—C3	−29 (2)
Tl1^vi^—O1—N1—C1	73.8 (9)	O3—N2—C2—C1	−175.4 (8)
Tl1^ii^—O1—N1—C1	37.9 (6)	Tl1^i^—N2—C2—C1	17.5 (11)
Tl1^vi^—O1—N1—Tl1	87.4 (6)	Tl1^ii^—N2—C2—C1	−44.7 (8)
Tl1^ii^—O1—N1—Tl1	51.6 (4)	Tl1^vii^—N2—C2—C1	−124.2 (7)
Tl1—O1—N1—Tl1^ii^	−51.6 (4)	Tl1^viii^—N2—C2—C1	154.6 (11)
Tl1^vi^—O1—N1—Tl1^ii^	35.9 (5)	O3—N2—C2—Tl1^ii^	−130.7 (7)
Tl1—O1—N1—Tl1^vi^	−87.4 (6)	Tl1^i^—N2—C2—Tl1^ii^	62.2 (4)
Tl1^ii^—O1—N1—Tl1^vi^	−35.9 (5)	Tl1^vii^—N2—C2—Tl1^ii^	−79.4 (5)
Tl1—O2—C1—N1	13.2 (13)	Tl1^viii^—N2—C2—Tl1^ii^	−160.6 (16)
Tl1^ii^—O2—C1—N1	−106.2 (10)	O3—N2—C2—Tl1^i^	167.1 (9)
Tl1^i^—O2—C1—N1	162.0 (7)	Tl1^ii^—N2—C2—Tl1^i^	−62.2 (4)
Tl1^vi^—O2—C1—N1	−38.7 (10)	Tl1^vii^—N2—C2—Tl1^i^	−141.7 (8)
Tl1^iv^—O2—C1—N1	79.1 (11)	Tl1^viii^—N2—C2—Tl1^i^	137.1 (18)
Tl1—O2—C1—C2	−170.2 (6)	O2—C1—C2—N2	−1.5 (14)
Tl1^ii^—O2—C1—C2	70.4 (8)	N1—C1—C2—N2	175.7 (9)
Tl1^i^—O2—C1—C2	−21.4 (12)	Tl1^ii^—C1—C2—N2	48.9 (9)
Tl1^vi^—O2—C1—C2	137.9 (7)	Tl1—C1—C2—N2	−18 (2)
Tl1^iv^—O2—C1—C2	−104.3 (8)	Tl1^i^—C1—C2—N2	−13.9 (9)
Tl1—O2—C1—Tl1^ii^	119.5 (6)	Tl1^vi^—C1—C2—N2	95.3 (11)
Tl1^i^—O2—C1—Tl1^ii^	−91.7 (7)	O2—C1—C2—C3	−176.8 (9)
Tl1^vi^—O2—C1—Tl1^ii^	67.6 (2)	N1—C1—C2—C3	0.4 (14)
Tl1^iv^—O2—C1—Tl1^ii^	−174.7 (6)	Tl1^ii^—C1—C2—C3	−126.4 (9)
Tl1^ii^—O2—C1—Tl1	−119.5 (6)	Tl1—C1—C2—C3	166.8 (11)
Tl1^i^—O2—C1—Tl1	148.8 (10)	Tl1^i^—C1—C2—C3	170.8 (10)
Tl1^vi^—O2—C1—Tl1	−51.9 (4)	Tl1^vi^—C1—C2—C3	−80.0 (13)
Tl1^iv^—O2—C1—Tl1	65.8 (5)	O2—C1—C2—Tl1^ii^	−50.4 (7)
Tl1—O2—C1—Tl1^i^	−148.8 (10)	N1—C1—C2—Tl1^ii^	126.8 (8)
Tl1^ii^—O2—C1—Tl1^i^	91.7 (7)	Tl1—C1—C2—Tl1^ii^	−66.7 (14)
Tl1^vi^—O2—C1—Tl1^i^	159.3 (7)	Tl1^i^—C1—C2—Tl1^ii^	−62.78 (17)
Tl1^iv^—O2—C1—Tl1^i^	−82.9 (6)	Tl1^vi^—C1—C2—Tl1^ii^	46.4 (7)
Tl1—O2—C1—Tl1^vi^	51.9 (4)	O2—C1—C2—Tl1^i^	12.4 (7)
Tl1^ii^—O2—C1—Tl1^vi^	−67.6 (2)	N1—C1—C2—Tl1^i^	−170.4 (8)
Tl1^i^—O2—C1—Tl1^vi^	−159.3 (7)	Tl1^ii^—C1—C2—Tl1^i^	62.78 (17)
Tl1^iv^—O2—C1—Tl1^vi^	117.8 (5)	Tl1—C1—C2—Tl1^i^	−3.9 (14)
O1—N1—C1—O2	1.8 (14)	Tl1^vi^—C1—C2—Tl1^i^	109.2 (8)
Tl1—N1—C1—O2	−8.4 (9)	O3—N3—C3—N4	−176.4 (8)
Tl1^ii^—N1—C1—O2	69.4 (9)	Tl1^vii^—N3—C3—N4	130.2 (8)
Tl1^vi^—N1—C1—O2	40.0 (11)	Tl1^viii^—N3—C3—N4	−162.5 (7)
O1—N1—C1—C2	−175.0 (8)	O3—N3—C3—C2	1.0 (10)
Tl1—N1—C1—C2	174.8 (8)	Tl1^vii^—N3—C3—C2	−52.4 (10)
Tl1^ii^—N1—C1—C2	−107.4 (9)	Tl1^viii^—N3—C3—C2	14.8 (11)
Tl1^vi^—N1—C1—C2	−136.8 (6)	Tl1^ix^—N4—C3—N3	29.1 (18)
O1—N1—C1—Tl1^ii^	−67.6 (9)	Tl1^ix^—N4—C3—C2	−147.8 (9)
Tl1—N1—C1—Tl1^ii^	−77.8 (4)	N2—C2—C3—N3	−1.1 (11)
Tl1^vi^—N1—C1—Tl1^ii^	−29.4 (5)	C1—C2—C3—N3	174.6 (9)
O1—N1—C1—Tl1	10.2 (7)	Tl1^ii^—C2—C3—N3	85.0 (10)
Tl1^ii^—N1—C1—Tl1	77.8 (4)	Tl1^i^—C2—C3—N3	−27.9 (19)
Tl1^vi^—N1—C1—Tl1	48.4 (2)	N2—C2—C3—N4	176.1 (9)
O1—N1—C1—Tl1^i^	36 (2)	C1—C2—C3—N4	−8.2 (16)
Tl1—N1—C1—Tl1^i^	26.0 (17)	Tl1^ii^—C2—C3—N4	−97.8 (11)
Tl1^ii^—N1—C1—Tl1^i^	103.8 (19)	Tl1^i^—C2—C3—N4	149.3 (12)

Symmetry codes: (i) −*x*, *y*+1/2, −*z*+3/2; (ii) −*x*, *y*−1/2, −*z*+3/2; (iii) *x*, −*y*+1/2, *z*+1/2; (iv) *x*, *y*+1, *z*; (v) *x*, −*y*+3/2, *z*+1/2; (vi) *x*, *y*−1, *z*; (vii) *x*, −*y*+1/2, *z*−1/2; (viii) *x*, −*y*+3/2, *z*−1/2; (ix) −*x*+1, *y*−1/2, −*z*+3/2.

### Hydrogen-bond geometry (Å, º)

**Table:**

*D*—H···*A*	*D*—H	H···*A*	*D*···*A*	*D*—H···*A*
N4—H4*A*···N3^x^	0.93	2.23	3.156 (10)	169
N4—H4*B*···N1^ix^	1.01	2.65	3.256 (13)	118

Symmetry codes: (ix) −*x*+1, *y*−1/2, −*z*+3/2; (x) −*x*+1, −*y*, −*z*+1.

## References

[bb1] Agilent (2012). *CrysAlis PRO*. Agilent Technologies Ltd, Yarnton, England.

[bb2] Akhbari, K., Alizadeh, K., Morsali, A. & Zeller, M. (2009). *Inorg. Chim. Acta*, **362**, 2589–2594.

[bb3] Akhbari, K. & Morsali, A. (2010). *Coord. Chem. Rev.* **254**, 1977–2006.

[bb4] Bourhis, L. J., Dolomanov, O. V., Gildea, R. J., Howard, J. A. K. & Puschmann, H. (2015). *Acta Cryst.* A**71**, 59–75. 10.1107/S2053273314022207 PMC4283469 25537389

[bb5] Britton, D. (2001). *Acta Cryst.* E**57**, m176–m178.

[bb6] De, S., Khambete, M. P. & Degani, M. S. (2019). *Bioorg. Med. Chem. Lett.* **29**, 1999–2007. 10.1016/j.bmcl.2019.06.054 31296357

[bb7] Dolomanov, O. V., Bourhis, L. J., Gildea, R. J., Howard, J. A. K. & Puschmann, H. (2009). *J. Appl. Cryst.* **42**, 339–341.

[bb8] Fershtat, L. L. & Makhova, N. N. (2016). *Russ. Chem. Rev.* **85**, 1097–1145.

[bb9] Fonari, M. S., Simonov, Yu. A., Kravtsov, V. Ch., Lipkowski, J., Ganin, E. V. & Yavolovskii, A. A. (2003). *J. Mol. Struct.* **647**, 129–140.

[bb10] Giorgis, M., Lolli, M. L., Rolando, B., Rao, A., Tosco, P., Chaurasia, S., Marabello, D., Fruttero, R. & Gasco, A. (2011). *Eur. J. Med. Chem.* **46**, 383–392. 10.1016/j.ejmech.2010.10.029 21109332

[bb11] Glomb, T., Szymankiewicz, K. & Świątek, P. (2018). *Molecules*, **23**, 3361–3377. 10.3390/molecules23123361 PMC6320996 30567416

[bb12] Golenya, I. A., Gumienna-Kontecka, E., Boyko, A. N., Haukka, M. & Fritsky, I. O. (2012). *Inorg. Chem.* **51**, 6221–6227. 10.1021/ic300387e 22607026

[bb13] Groom, C. R., Bruno, I. J., Lightfoot, M. P. & Ward, S. C. (2016). *Acta Cryst.* B**72**, 171–179. 10.1107/S2052520616003954 PMC4822653 27048719

[bb14] Gumienna-Kontecka, E., Golenya, I. A., Dudarenko, N. M., Dobosz, A., Haukka, M., Fritsky, I. O. & Swiatek-Kozlowska, J. (2007). *New J. Chem.* **31**, 1798–1805.

[bb15] Kumar, S., Sharma, R. P., Saini, A., Venugopalan, P. & Starynowicz, P. (2015). *J. Mol. Struct.* **1079**, 291–297.

[bb17] Larsen, I. K. (1988). *Acta Cryst.* B**44**, 527–533.

[bb18] Lutter, J. C., Zaleski, C. M. & Pecoraro, V. L. (2018). *Adv. Inorg. Chem.* 177–246.

[bb19] Mudring, A. V. & Rieger, F. (2005). *Inorg. Chem.* **44**, 6240–6243. 10.1021/ic050547k 16124801

[bb20] Neel, A. J. & Zhao, R. (2018). *Org. Lett.* **20**, 2024–2027. 10.1021/acs.orglett.8b00568 29553273

[bb21] Ostrowska, M., Golenya, I. A., Haukka, M., Fritsky, I. O. & Gumienna-Kontecka, E. (2019). *New J. Chem.* **43**, 10237–10249.

[bb22] Pal, P., Gandhi, H. P., Kanhed, A. M., Patel, N. R., Mankadia, N. N., Baldha, S. N., Barmade, M. A., Murumkar, P. R. & Yadav, M. R. (2017). *Eur. J. Med. Chem.* **130**, 107–123. 10.1016/j.ejmech.2017.02.038 28242547

[bb23] Pavlishchuk, A. V., Kolotilov, S. V., Zeller, M., Lofland, S. E. & Addison, A. W. (2018). *Eur. J. Inorg. Chem.* pp. 3504–3511.

[bb24] Pibiri, I., Lentini, L., Melfi, R., Tutone, M., Baldassano, S., Galluzzo, P. R., Di Leonardo, A. & Pace, A. (2018). *Eur. J. Med. Chem.* **159**, 126–142. 10.1016/j.ejmech.2018.09.057 30278331

[bb25] Robertson, D., Barnes, C. & Gerasimchuk, N. (2004). *J. Coord. Chem.* **57**, 1205–1216.

[bb26] Romeo, G. & Chiacchio, U. (2011). *Modern Heterocyclic Chemistry*, edited by J. Alvarez-Builla, J. J. Vaquero & J. Barluenga, pp. 1047–1252. Weinheim: Wiley-VCH.

[bb27] Safyanova, I. S., Ohui, K. A. & Omelchenko, I. V. (2017). *Acta Cryst.* E**73**, 24–27. 10.1107/S2056989016019095 PMC5209763 28083127

[bb28] Salassa, G. & Terenzi, A. (2019). *Int. J. Mol. Sci.* **20**, 3483–3500. 10.3390/ijms20143483 PMC6678488 31315181

[bb29] Schroffenegger, M., Eder, F., Weil, M., Stöger, B., Schwendtner, K. & Kolitsch, U. (2020). *J. Alloys Compd.* **820**, 153369.

[bb30] Shaposhnikov, S., Pirogov, S. V., Mel’nikova, S. F., Tselinsky, I. V., Näther, C., Graening, T., Traulsen, T. & Friedrichsen, W. (2003). *Tetrahedron*, **59**, 1059–1066.

[bb31] Sheldrick, G. M. (2015). *Acta Cryst.* A**71**, 3–8.

[bb16] Singh, A. K., Lohani, M. & Parthsarthy, R. (2013). *Iran. J. Pharm. Res.* **12**, 319–323. PMC3813233 24250606

[bb32] Stepanov, A. I., Astrat’ev, A. A., Sheremetev, A. B., Lagutina, N. K., Palysaeva, N. V., Tyurin, A. Yu., Aleksandrova, N. S., Sadchikova, N. P., Suponitsky, K. Yu., Atamanenko, O. P., Konyushkin, L. D., Semenov, R. V., Firgang, S. I., Kiselyov, A. S., Semenova, M. N. & Semenov, V. V. (2015). *Eur. J. Med. Chem.* **94**, 237–251. 10.1016/j.ejmech.2015.02.051 25768706

[bb33] Telvekar, V. N. & Takale, B. S. (2013). *Synth. Commun.* **43**, 221–227.

[bb34] Viterbo, D. & Serafino, A. (1978). *Acta Cryst.* B**34**, 3444–3446.

[bb35] Wiesbrock, F. & Schmidbaur, H. (2003). *J. Am. Chem. Soc.* **125**, 3622–3630. 10.1021/ja0287783 12643725

[bb36] Wiesbrock, F. & Schmidbaur, H. (2004). *J. Inorg. Biochem.* **98**, 473–484. 10.1016/j.jinorgbio.2003.12.017 14987848

[bb37] Zareef, M. I., Iqbal, R., De Dominguez, N. G., Rodrigues, J., Zaidi, J. H., Arfan, M. & Supuran, C. T. (2007). *J. Enzyme Inhib. Med. Chem.* **22**, 301–308. 10.1080/14756360601114569 17674812

[bb38] Zhang, H. & Jian, F. (2009). *Acta Cryst.* E**65**, o2911. 10.1107/S1600536809044432 PMC2971256 21578490

[bb39] Zhang, J., Mitchell, L. A., Parrish, D. A. & Shreeve, J. M. (2015). *J. Am. Chem. Soc.* **137**, 10532–10535. 10.1021/jacs.5b07852 26262555

[bb40] Zlotin, S. G., Churakov, A. M., Dalinger, I. L., Luk’yanov, O. A., Makhova, N. N., Sukhorukov, A. Yu. & Tartakovsky, V. A. (2017). *Mendeleev Commun.* **27**, 535–546.

